# The German version of the mHealth App Usability Questionnaire (GER-MAUQ): Translation and validation study in patients with cardiovascular disease

**DOI:** 10.1177/20552076231225168

**Published:** 2024-01-31

**Authors:** Theodora Tacke, Pascal Nohl-Deryk, Neelam Lingwal, Lara Marie Reimer, Fabian Starnecker, Corina Güthlin, Ferdinand M Gerlach, Heribert Schunkert, Stephan Jonas, Angelina Müller

**Affiliations:** 1 Institute of General Practice, 9173 Goethe University Frankfurt, Frankfurt am Main, Germany; 2 Institute of Biostatistics and Mathematical Modeling, Goethe University, Frankfurt, Germany; 3 School of Computation, Information and Technology, 9184 Technical University of Munich, Munich, Germany; 4 Institute for Digital Medicine, 39062 University Hospital Bonn, Bonn, Germany; 5 Department of Cardiology, German Heart Center Munich, 9184 Technical University of Munich, Munich, Germany; 6 Deutsches Zentrum für Herz- und Kreislauferkrankungen (DZHK), Partner Site Munich Heart Alliance, Munich, Germany; 7 Medical Graduate Center, 9184 Technical University of Munich, Munich, Germany

**Keywords:** mHealth app, usability, evaluation tool, questionnaire, questionnaire validation, German version of MAUQ, German language, validity, reliability, mHealth

## Abstract

**Objective:**

In Germany, only a few standardized evaluation tools for assessing the usability of mobile Health apps exist so far. This study aimed to translate and validate the English patient version for standalone apps of the mHealth App Usability Questionnaire (MAUQ) into a German version.

**Methods:**

Following scientific guidelines for translation and cross-cultural adaptation, the patient version for standalone apps was forward and back-translated from English into German by an expert panel. In total, 53 participants who were recruited as part of the beta testing process of the recently developed mHealth app HerzFit, answered the questions of the German version of the MAUQ (GER-MAUQ) and the System Usability Scale. Subsequently, a descriptive as well as a psychometric analysis was performed to test validity and reliability.

**Results:**

After conducting three cognitive interviews, five items were modified. The values for Cronbach alpha for the entire questionnaire and the three subscales (0.966, 0.814, 0.910, and 0.909) indicate strong internal consistency. The correlation analysis revealed that the scores of the GER-MAUQ, the subscales and the SUS were strongly correlated with each other. The correlation coefficient of the SUS and the GER-MAUQ overall score was r = 0.854, P < 0.001 and the coefficients of the subscales and the SUS were r = 0.642, P < 0.001; r = 0.866, P < 0.001 and r = 0.643, P < 0.001.

**Conclusions:**

We have developed a novel German version of the MAUQ and demonstrated it as a reliable and valid measurement tool for assessing the usability of standalone mHealth apps from the patients’ perspective. The GER-MAUQ allows a new form of standardized assessment of usability of mHealth apps for patients with cardiovascular disease in Germany. Further research with a larger sample and other samples is recommended.

## Introduction

Mobile health (mHealth) applications can contribute greatly to strengthening the health care system worldwide: They can support medical staff in the treatment of various medical conditions, facilitate patients' health self-management, prevent a variety of different diseases and thus save health care system costs.^1–5^ As the first country in the world to do so, Germany offers patients the right to reimbursement of the costs of mHealth apps through statutory health insurance.^6^ The interest in mHealth applications is growing in health care systems around the world. As the number of mHealth apps increases, the demand for scientific evaluation of these services is strongly increasing as well.^7,8^ Despite the growing popularity of mHealth applications, the intensity of reporting usability results does not correlate with the number of reported eHealth implementation studies.^9,10^

Usability is the major quality factor of such apps and determines whether patients continue to use an app or cease to use it.^11–13^ According to the International Organization of Standardization (ISO) 9241-11, usability is the “extent to which a system, product or service can be used by specified users to achieve specified goals with effectiveness, efficiency and satisfaction in a specified context of use”.^14^ The primary reasons for discontinuing mHealth apps are high data entry burdens, data privacy concerns, loss of interest and hidden costs.^13^ In order to appropriately gear mHealth apps to their target users, a reliable usability assessment and standardized methods are required.

The most commonly used method for assessing the usability of electronic as well as mobile health technologies is the questionnaire.^9^ General and technology-independent questionnaires such as the System Usability Scale (SUS) and the Post-Study System Usability Questionnaire (PSSUQ) are commonly employed in usability studies of mHealth apps.^15–17^ However, these questionnaires were created for general software systems and cannot reliably identify mHealth-specific problems that may arise, for example, in patients’ health self-management or in accessing health care services. In addition, these questionnaires were not validated by the target users of mHealth apps, such as patients, but by other populations.^15,17^ Therefore, many app developers design their individual questionnaires and conduct the validation with only a small number of study participants.^10,18^

Previous usability assessments of mHealth apps are substandard according to the ISO definition, and researchers should prefer using well-validated questionnaires such as the mHealth App Usability Questionnaire (MAUQ) for their usability analysis.^10,19^ The MAUQ is currently the gold standard and designed specifically for mHealth apps^10^; it considers its target users (patients or providers) as well as the type of app (interactive or standalone).^19^ In recent years, the MAUQ has been translated from the original English into several other languages and used in numerous usability studies.^20–22^

There are only a few validated questionnaires available for evaluation of mHealth apps in English-speaking countries.^16^ So far, there are only a limited number of standardized German-language questionnaires at all for assessing the usability of mHealth apps from the patients’ perspective as target users.^23,24^

These facts indicate the need for more validated and standardized questionnaires to evaluate mHealth app usability, and especially for a version in German.

To counteract the lack of any German-language questionnaires in the field of usability assessment, we have translated the MAUQ into German and validated it with a standardized questionnaire. In so doing, we aimed to create a high-quality German evaluation tool for future research and thereby contribute to the improvement of digital patient care in other German-speaking countries.

## Methods

### Overview of the MAUQ

The mHealth App Usability Questionnaire is a validated and reliable English-language questionnaire designed by Zhou et al. to specifically assess the usability of mHealth apps. Authors’ consent to translate and validate the MAUQ into German was obtained. The MAUQ was developed based on 38 selected existing usability questionnaires, which included well-validated questionnaires such as SUS, Post-Study System Usability Questionnaire and Perceived Usefulness and Ease of Use. Overall, these surveys cover various aspects of user experience and usability. There are a total of four versions: two for standalone and two for interactive mHealth apps, one of each for patients and one of each for providers, as target users.^25^ The patient version for standalone apps used in this study consists of 18 questions that can be divided into three subscales: ease of use (five items), interface and satisfaction (seven items) and usefulness (six items). Cronbach alpha for this version is 0.914, demonstrating strong internal consistency. The study participants rated the questions on a 7-point Likert scale from 1 (disagree) to 7 (agree) or alternatively chose “not to answer”. In the data analysis, missing data were replaced with the value of 4. The overall average was calculated by determining the sum and the average of all responses, applying the following rule: The higher the overall average, the greater the usability.^19^

### Overview of the SUS

The System Usability Scale, or SUS, is a commonly used questionnaire by J. Brooke.^16,17^ The questionnaire is easy to understand and reliably measures the usability of general systems. The SUS consists of 10 questions, one half of which are worded positively and one half negatively. The 10 items are rated on a five-point Likert scale between 1 (=strongly agree) and 5 (=strongly disagree). Responses are coded 0 to 4, depending on the negative or positive wording. The SUS score is calculated by adding the individual scores and then multiplying that sum by 2.5. The result is a score between 0 and 100, where 100 is the best possible score. The SUS has already been translated and validated in many languages.^26^ Individual problems that patients may have when using mHealth apps are not specifically identified by the SUS.

### Translation process and cultural adaptation

A panel of experts translated all four original versions of the MAUQ questionnaire from English into German in accordance with scientific guidelines for translation and cross-cultural adaptation.^27,28^ Since this study focuses on the validation of the patient version for standalone mHealth apps, only this version was queried in cognitive interviews.

**Figure A1.**
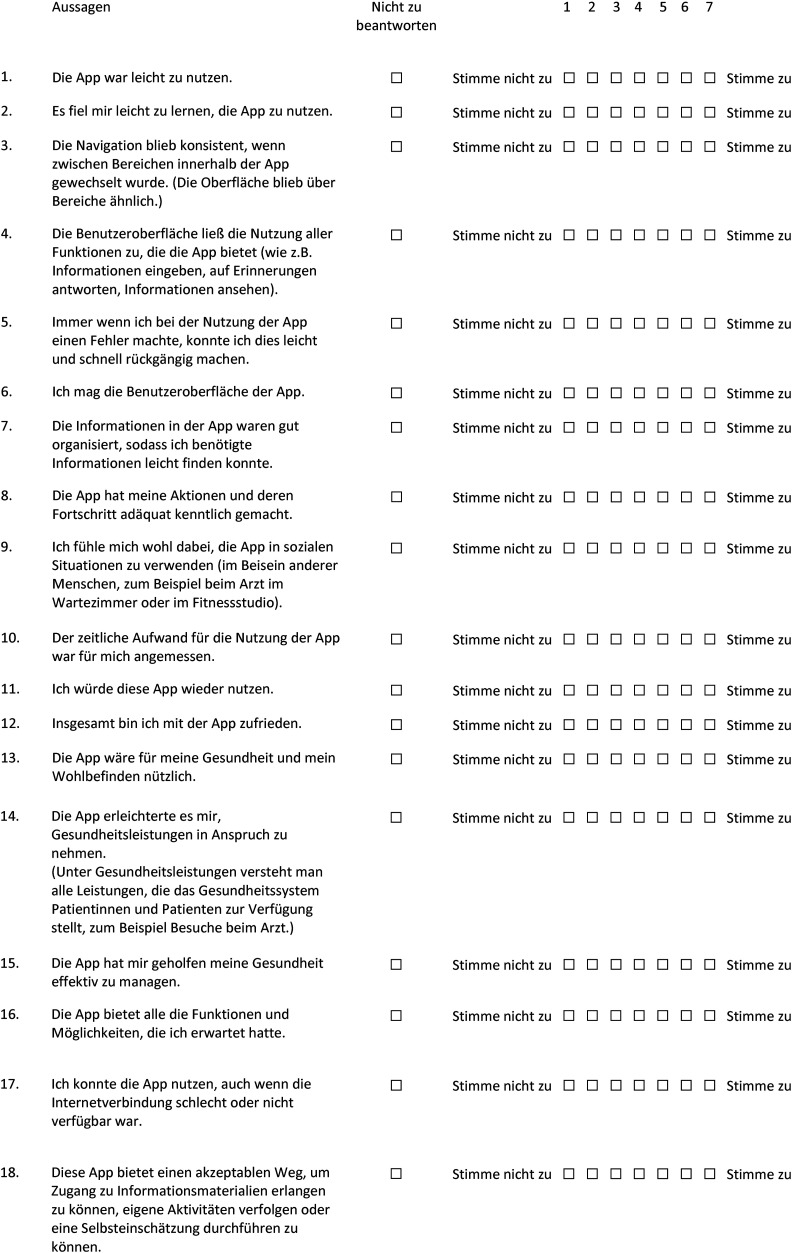
The German version of the mHealth App Usability Questionnaire.

The process of translation can be divided into five steps. Step 1: Forward translation: In the first step, A.M. and P.N., who are native German speakers proficient in English produced independent translations of the original MAUQ. A.M. and P.N. are physicians in the field of general medicine and have profound experience in the development of questionnaires and patient surveys. Step 2: Expert panel: The translations were discussed by an expert panel that included C.G., A.M. and P.N. and two other medical scientists, all experienced in questionnaire development. The expert panel identified inadequate expressions and discrepancies between the translations. As a result, the first two translated versions were harmonized into an initial complete translation. Step 3: Back-translation. Two different native English speakers independently back-translated the original questionnaire as the next step. In a subsequent MAUQ consensus conference, a small group of six experts, including C.G., A.M. and P.N., then discussed the back-translation. They adjusted, reworded and improved on item discrepancies in the back-translation. Thus, a second version of the translated questionnaire was produced. Step 4: Pre-testing and cognitive interviewing: The reason for the following design of cognitive interviews is the fact that there is no general consensus in the realm of scientific discourse regarding an appropriate sample size, criteria for participant selection, the optimal qualifications and training standards for interviewers and the selection of appropriate cognitive probes.^29^ Based on scientific recommendations, T.T. and C.G., who has previous experience conducting cognitive interviews for other projects with questionnaires, created a protocol using the think-aloud method for the subsequent procedure.^30–32^ In a final step, T.T. reviewed the version for standalone apps for patients in cognitive interviews on three individuals with a profile similar to that of the selected mHealth app's actual target user: The target users of the selected app, which will be explained in more detail in the following section, are mainly middle-aged people with or at risk for cardiovascular disease and people who are interested in monitoring their health profile on their smartphone. For this reason, two people with chronic cardiovascular disease and one person without were selected. The participants were between 52 and 63 years old, owned their own smartphone, and were personally interested in the app and what it offered. All were native German speakers. The cognitive interviews were held as face-to-face conversations. Before completing the questionnaire, all three participants tested the selected mHealth app on a smartphone. The device was an iPhone 8 with Retina IPS LCD display, 4.7-inch (diagonal) screen with a 1334-by-750-pixel resolution at 326 pp. It contains the Apple A11 Bionic chip, which is a 64-bit ARM-based system.^33^ During pre-testing, participants were asked to read aloud the questions on the translated MAUQ questionnaire, to express their thoughts about questions and personal answers, to point out ambiguous or unknown terms and to make suggestions for alternative wording. The pre-testing was aimed at gaining insight into the respondents’ understanding and response strategy. The participants were not interrupted by the interviewer during the thinking process. T.T. asked specific questions for items 3, 8, 9, 14, and 18: For example, in item 9 “I feel comfortable in social settings”, participants were queried about their understanding of the term “social settings”. The interviews were recorded with an external technical device and T.T. documented the participants’ observations and remarks in note form. The raw data consisted of the responses from the second version of the translated questionnaire and T.T.'s notes and quotes. A qualitative content analysis of the interviews followed, focusing on the identification of the items that seemed difficult to understand.^31^ After analyzing the three interviews, it was decided that due to the congruent results, conducting a total of three interviews was sufficient. Step 5: Final version: Based on the results of the pre-testing, a final version of the translated MAUQ questionnaire was created by C.G. P.N. and T.T. This version was called the German mHealth App Usability Questionnaire, GER-MAUQ for short, and corresponds to the German translation of the patient version of the mHealth App Usability Questionnaire for standalone mHealth apps. The complete questionnaire can be found in the Multimedia Appendix (Figure A1).

### Study design and setting

#### The mHealth app used for validation of the GER-MAUQ

The validation of the GER-MAUQ was conducted using the HerzFit app, which was developed within the framework of DigiMed Bayern, the flagship project to advance digital medicine in Bavaria, under the direction of the German Heart Centre and the School of Computation, Information and Technology of the Technical University of Munich.^34^ The HerzFit app is a lifestyle app aimed at improving the prevention of cardiovascular diseases and providing guidance and support for its users in their everyday lives. HerzFit was developed together with the German Heart Foundation (the largest, non-profit patient advocacy group in the field of heart disease in Europe (>100,000 members))^35^ and the German Hypertension League.^36^ The app is operated by the German Heart Foundation and is available for free in Germany, Austria and Switzerland. HerzFit is a standalone app since communication between the user and health care provider is not possible.

#### Study participants

The study participants of this validation study were recruited through the channels of the German Heart Foundation and the German Hypertension League as part of the beta testing phase of HerzFit. Study participants were mainly people who have a cardiovascular disease or who are in close contact with people suffering from a cardiovascular disease. Therefore, the members were deemed to be representative of HerzFit's potential target group. Study participation was voluntary and a declaration of consent was required. Exclusion criteria were not determined except for the possession of a smartphone/tablet with the downloaded HerzFit app. All questions had to be answered in order for the results to be included in the analysis.

#### Sample size

We estimated the sample size using the BiAs program^37^, based on the correlation coefficients of the scales and subscales calculated in the original study of the MAUQ. We chose the correlations between the subscale “usefulness” and the overall scale of the MAUQ, respectively, with the scale of the SUS as primary outcome variable. We included a significance correction and chose a significance level of α = 0.025. The result showed that at least 55 patients should be included in the study to collect the statistically significant results regarding the validity analysis.

#### Usability study and research tools

First, members of the self-help groups were recruited. In addition, a public announcement was posted on the German Heart Foundation's social media channels, allowing anyone interested to sign up. Employees of the two cooperating patient foundations were excluded. Information about the study and a declaration of consent was sent to all interested persons. Subsequently, the study participants who returned the declaration of consent were invited to the testing phase of the HerzFit app (version 0.9.101) for a duration of seven days. No specific instructions on how to use the app were given. After this period, the link to the survey portal was sent to all study participants via email. LimeSurvey software was used to provide the online survey.^39^ Before completing the online questionnaire, the study participants received a brief explanation of the study process and goal. It was also emphasized that the data would be stored in a strictly confidential and anonymous manner.

**Figure A2.**
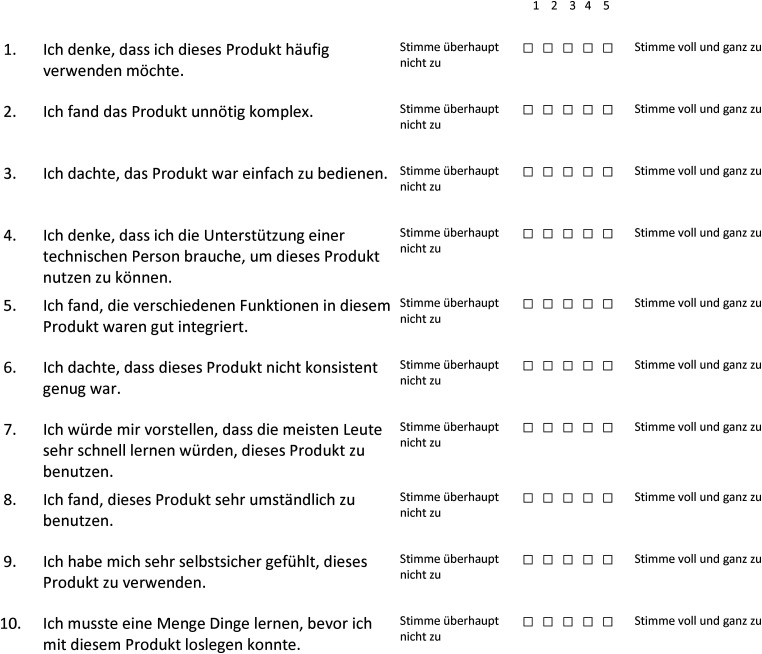
The German translation of the System Usability Scale.

The online questionnaire contained three parts. The first part asked for information about the participant themself, including age, gender, marital status, general level of completed education, highest professional degree, place of residence and employment. The first part additionally included one question about the use of apps on the smartphone/tablet and one question about the use of technical devices that record one's performance. Moreover, participants were asked to indicate how often they had used the HerzFit app in the last seven days. The second part is followed with the 18 questions of the GER-MAUQ and then the third part, consisting of the 10 questions of the validated German translation of the SUS (Figure A2).^26^

### Statistical methods

#### Descriptive analysis

A total of 57 electronic invitations were sent via email with access to the online questionnaire. Fifty-three questionnaires were included in the study and four questionnaires were excluded due to incomplete replies. The answer option “not able to answer” of the GER-MAUQ was evaluated as missing data in the analysis. We verified the primary outcome measure, the correlation analysis, as well as the reliability analysis also by using a value of 4 for missing data. The participants’ responses ranged from 1 (strongly disagree) to 7 (agree) for the GER-MAUQ, and from 1 (strongly disagree) to 5 (strongly agree) for the SUS.

A descriptive analysis was performed to obtain an overview of study participant demographic data and of the overall outcome. In this step, the GER-MAUQ subscale scores, the GER-MAUQ overall score, and the means and standard deviations were calculated. For analysis of the SUS, the overall score was determined using the standard score conversion procedure and converted to a value between 0 and 100.

#### Data distribution

The demographic data, the scales of the SUS and GER-MAUQ, and its subscales, were tested for normal distribution using the Kolmogorov-Smirnov test and the Shapiro-Wilk test. The data were considered normally distributed if P value > 0.05 in one of the two tests.

#### Demographic characteristics

We evaluated the impact of the demographic factors on the individual responses and the overall score of the GER-MAUQ. For non-normal distribution, we used the Mann-Whitney U test for comparisons between two groups and the Kruskal-Wallis test for comparisons between three or more groups. If the Kruskal-Wallis test showed a statistically significant difference between the groups, pairwise comparisons were performed using the Dunn-Bonferroni test.

#### Validity and reliability testing

The statistical data analysis was performed using SPSS version 29.0.0.0**.** First, an item analysis was carried out. The aim of the item analysis was to determine whether any items of the GER-MAUQ should be removed from the questionnaire. To examine the differences in central tendency of the items 1–18, Mann-Whitney U tests were conducted. Therefore, the participants were divided into a low-score and a high-score group, following percentiles data grouping in the Chinese MAUQ validation study. If the overall GER-MAUQ score was below the 27th percentile (≤3.70199), the respective participant was assigned to the low-score group. If above the 73th percentile (≥5.5000), the participant was assigned to the high-score group.^40^ P < 0.05 indicated a statistically significant difference between the mean scores of the items, representing acceptable differentiation between the low- and high-score groups. Subsequently, the items were correlated with the overall GER-MAUQ score. For normally distributed data, the Pearson correlation analysis was conducted and for non-normally distributed data the Spearman correlation.

To examine the validity of the translated questionnaire, the correlation coefficients between the overall score of the GER-MAUQ, the scores of the three subscales and the score of the SUS were determined. Again, Pearson correlation was used for normally distributed data and Spearman correlation for non-normally distributed data. Cronbach alpha was calculated for each question in order to assess the internal consistency of the questionnaire. A high value for Cronbach alpha indicates good reliability. In research studies, Cronbach alpha values ranging from 0.7 to 0.8 are deemed acceptable, while a value of approximately 0.9 is considered excellent.^41^

## Results

### Results of the cognitive interviews

According to the results of the cognitive interviews and the participants’ comments, five questions of the GER-MAUQ were modified to improve comprehensibility, reading flow and language expression. As item 3 “The navigation was consistent when moving between screens” was not comprehensible, the following was added: “The interface remained similar across areas.” The sentence structure of item 4 disrupted reading flow, so the part “such as entering information, responding to reminders, viewing information” was placed at the end of the sentence. The term “social settings” in item 9 was not clearly understandable in German, thus the following explanation was added in brackets: “in the presence of other people, for example, in the waiting room at the doctor's or in the gym”. In order to make item 14 “The app improved my access to health care services” clearer for all participants, the term “health care services” was explained as follows: “Health care services are all services that the health care system offers patients, for example, visits to the doctor”. Item 18 “This mHealth app provided an acceptable way to receive health care services, such as accessing education materials, tracking my own activities and performing self-assessment” was changed to “This app provided an acceptable way to access information materials, perform self-assessment and track my own activities.”

### Study participants

A similar number of women (26) and men (27) participated in the survey. The majority of the participants were between 50 and 69 years old (71.7%). Almost three-quarters of the participants were married (73.6%) and more than half of the participants had the highest general school qualification – high school graduation (56.6%). In the category ‘highest professional qualification’, almost half of the participants stated that they had completed recognized vocational training (49.1%). No participant preferred analog options such as books, dictionaries, journals, and pocket diaries over smartphone/tablet apps (0.0%). Of the participants, 58.5% decided on a case-by-case basis whether to choose apps or analog options. 83% of the participants suffered from chronic cardiovascular diseases for which they regularly take medication. In addition, over 90% of respondents had experience with a technical device, such as a wristband with a pedometer, which records physical performance. Further characteristics of the study participants are summarized in Table 1.

**Table 1.** Demographic characteristics of the study participants (n = 53).

Characteristics		Participants, n (%)
Age (in years)		
	< 20	0 (0.0)
	20–29	2 (3.8)
	30–39	4 (7.5)
	40–49	4 (7.5)
	50–59	17 (32.1)
	60–69	21 (39.6)
	70–79	4 (7.5)
	> 80	1 (1.9)
Gender		
	Man	27 (50.9)
	Woman	26 (49.1)
	Non-binary	0 (0.0)
Marital status		
	Single	8 (15.1)
	Married	39 (73.6)
	Divorced	4 (7.5)
	Widowed	1 (1.9)
	Registered civil partnership	1 (1.9)
	Dissolved civil partnership	0 (0.0)
	Civil partner deceased	0 (0.0)
Highest level of education		
	No school certificate	1 (1.9)
	Elementary/secondary school certificate	9 (17.0)
	Intermediate school certificate	13 (24.5)
	High school certificate	30 (56.6)
Highest professional qualification		
	Still in vocational training	0 (0.0)
	No vocational qualification	3 (5.7)
	Recognized vocational training	26 (49.1)
	Master/technician/equivalent qualification	4 (7.5)
	Bachelor's degree	7 (13.2)
	Diploma/master's/magister degree/state examination	10 (18.9)
	PhD degree	3 (5.7)
Place of residence		
	Major city (>100,000)	16 (30.2)
	Large town (20,000–100,000)	12 (22.6)
	Small town (5000–20,000)	12 (22.6)
	Village (<5000)	13 (24.5)
Employment		
	Still studying	0 (0.0)
	Employed	30 (56.6)
	Retired	19 (35.8)
	Disabled	3 (5.7)
	Unemployed	1 (1.9)
Behavior in using apps or analog options		
	Preference for using analog options	0 (0.0)
	Preference for using apps	22 (41.5)
	Case-by-case decision	31 (58.5)
Previous usage of a technical device for measuring physical activity^a^		
	Previously used a technical device	48 (90.6)
	Never used a technical device	5 (9.4)
Chronic cardiovascular disease		
	With disease	44 (83.0)
	Without disease	9 (17.0)
Number of days using the HerzFit app^b^ in the previous 7 days		
	0–1 day	1 (1.8)
	2–4 days	11 (19.6)
	5–7 days	41 (73.2)

^a^ E.g. wristband/ chest strap with heart rate monitor or pedometer.

^b^ An app used to validate the German translation of the mHealth App Usability Questionnaire.

### Descriptive data

**Figure 1.**
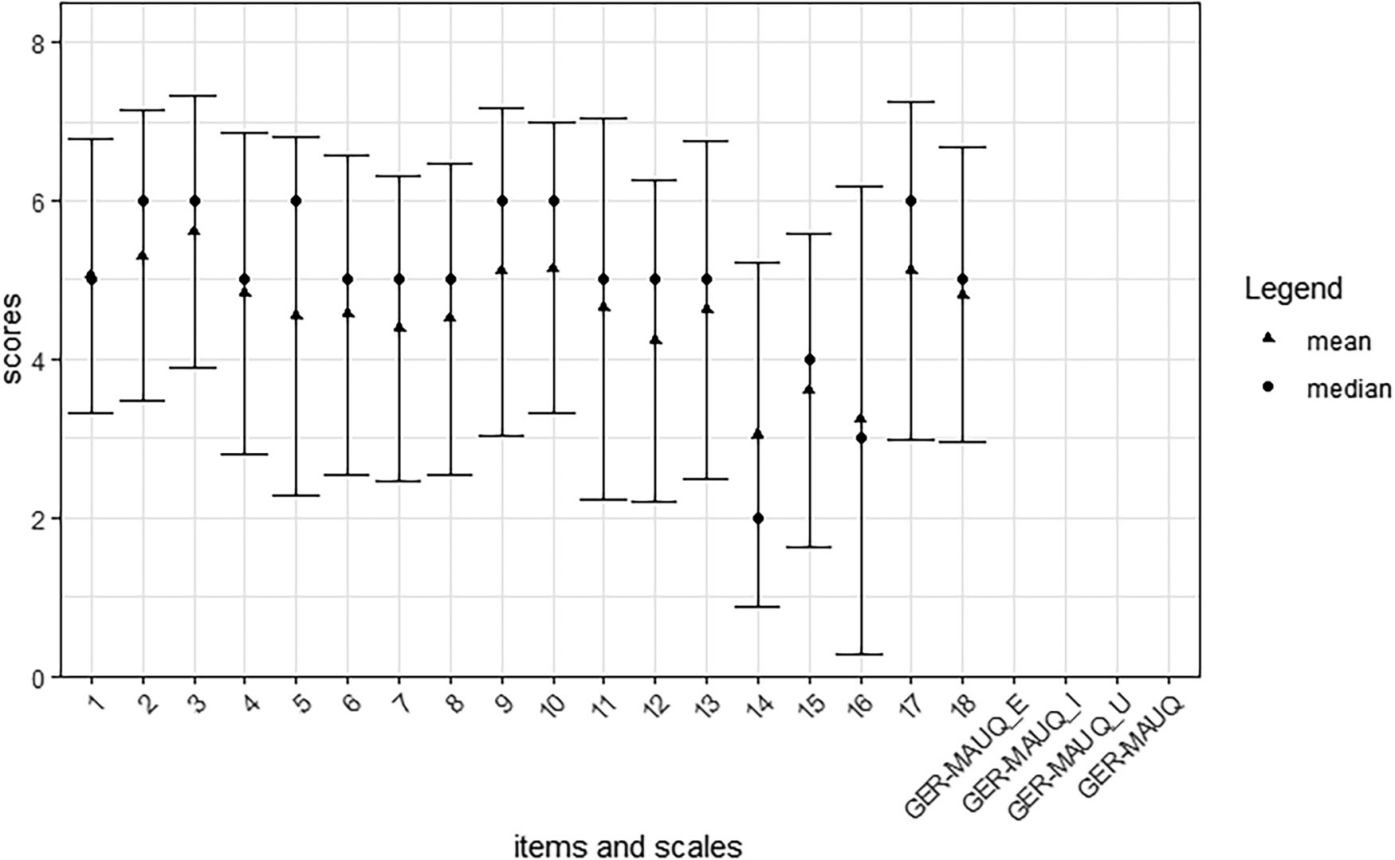
Descriptive statistics of the items and scale values of the GER-MAUQ.

Table 2 and Figure 1 show descriptive statistics for items 1–18 of the GER-MAUQ, the subscales of the GER-MAUQ, the overall score of the GER-MAUQ and of the SUS. As the answer option “not to be answered” of the GER-MAUQ was evaluated as missing data in the analysis, the number of participants (n) varies between items 1–18. Especially for item 9 and item 17, the quantity of missing data is high, which means many participants were not able to answer these questions related to the HerzFit app. Table A1 which appears in the Appendix shows the descriptive statistics when using the value 4 for missing data.

**Table 2.** Descriptive statistics of the items and scale values for the GER-MAUQ and SUS.

	n^a^	Median (range)	Mean (SD)
Items^b^			
1	53	5 (1–7)	5.06 (1.726)
2	52	6 (1–7)	5.31 (1.832)
3	52	6 (1–7)	5.62 (1.717)
4	51	5 (1–7)	4.84 (2.024)
5	46	6 (1–7)	4.54 (2.268)
6	51	5 (1–7)	4.57 (2.013)
7	52	5 (1–7)	4.40 (1.923)
8	49	5 (1–7)	4.51 (1.959)
9	37	6 (1–7)	5.11 (2.065)
10	52	6 (1–7)	5.15 (1.830)
11	50	5 (1–7)	4.64 (2.397)
12	52	5 (1–7)	4.23 (2.025)
13	53	5 (1–7)	4.62 (2.132)
14	43	2 (1–7)	3.05 (2.160)
15	51	4 (1–7)	3.61 (1.971)
16	50	3 (1–7)	3.24 (2.954)
17	25	6 (1–7)	5.12 (2.128)
18	52	5 (1–7)	4.81 (1.858)
Scale			
GER-MAUQ_E	53	5.2 (1–7)	5.077 (1.462)
GER-MAUQ_I	53	4.857 (1–7)	4.562 (1.659)
GER-MAUQ_U	53	4 (1–6.8)	4.014 (1.637)
GER-MAUQ	53	4.643 (1.167- 6.765)	4.533 (1.406)
German SUS	53	70 (22.5–97.5)	67.5 (18.779)

GER-MAUQ: German translation of the mHealth App Usability Questionnaire, overall score; GER-MAUQ_E: German translation of the mHealth App Usability Questionnaire, subscale: ease of use; GER-MAUQ_I: German translation of the mHealth App Usability Questionnaire, subscale: interface and satisfaction; GER-MAUQ_U: German translation of the mHealth App Usability Questionnaire, subscale: usefulness; SD: standard deviation; SUS: System Usability Scale.

^a^ Number of participants who rated item on the Likert scale.

^b^ Items 1–18 represent the 18 items of the German translation of the mHealth App Usability Questionnaire.

The subscales GER-MAUQ_I and GER-MAUQ_U, as well as the overall score of the GER-MAUQ and the SUS, were normally distributed according to the Kolmogorov-Smirnov test (P value > 0.05 in all cases). The distribution of the demographic data, the subscale GER-MAUQ_E and the distribution of most of the GER-MAUQ items were not normal according to the Kolmogorov-Smirnov test and the Shapiro-Wilk test (P value < 0.05 in all cases, except for items 4, 10, 12, 14, 16).

### Item analysis of the GER-MAUQ

Table 3 shows the results of the Mann-Whitney U test. The results of the item analysis show that there was a statistically significant difference between the mean scores of all items, except for item 4. This indicates that, in this case, low and high-score groups cannot be well distinguished from each other. As presented in Table 4, all items were correlated with the overall GER-MAUQ score. The correlation coefficient was not below 0.3 in any of the cases. Items 3 and 4 correlated moderately with the overall score, while all other items correlated strongly. All items presented a statistical significance concerning the correlation.

**Table 3.** Results of the item analysis.

	Group, median					
Items^a^	Low-score group	n	High-score group	n	Mann-Whitney U	P value^b^
1	3.5	14	7	15	31.500	<0.001
2	3	13	7	15	24.000	<0.001
3	5.5	14	7	15	60.500	0.036
4	4	12	6	15	65.500	0.219
5	3	13	7	14	14.500	<0.001
6	2	13	7	14	1.000	<0.001
7	1	13	6	15	6.000	<0.001
8	1.5	12	6	15	16.000	<0.001
9	1	9	6	12	14.000	0.003
10	3	13	7	15	31.500	0.002
11	1	11	7	15	9.000	<0.001
12	1	13	6	15	1.500	<0.001
13	2	14	7	15	32.500	<0.001
14	1	12	6	11	0.000	<0.001
15	1	12	5	15	0.000	<0.001
16	1	12	5	14	0.500	<0.001
17	1.5	6	6	8	3.500	0.006
18	2	13	6	15	9	<0.001

^a^The items 1–18 represent the 18 items of the German translation of the mHealth app Usability Questionnaire.

^b^Two-tailed P value.

**Table 4.** Results of the correlation analysis between each item and the overall score of the GER-MAUQ.

	Correlation coefficient^a^	P value^b^	n
GER-MAUQ^c^	1	-	53
Items^d^			
1	0.595	<0.001	53
2	0.607	<0.001	52
3	0.351	0.011	52
4^c^	0.424	0.002	51
5	0.647	<0.001	46
6	0.858	<0.001	51
7	0.743	<0.001	52
8	0.713	<0.001	49
9	0.624	<0.001	37
10^c^	0.683	<0.001	52
11	0.791	<0.001	50
12	0.860	<0.001	52
13	0.592	<0.001	53
14^c^	0.729	<0.001	43
15	0.869	<0.001	51
16^c^	0.730	<0.001	50
17	0.572	0.003	25
18	0.695	<0.001	52

GER-MAUQ: German translation of the mHealth App Usability Questionnaire, overall score.

^a^The correlation method based on the distribution of the data. For normally distributed data, the Pearson correlation analysis was conducted and for non-normally distributed data the Spearman correlation.

^b^Two-tailed P value.

^c^The data are normally distributed.

^d^The items 1–18 represent the 18 items of the German translation of the mHealth app Usability Questionnaire.

### Psychometric analysis results

#### Internal consistency

The results of the reliability testing are presented in Table 5. The values of Cronbach alpha show that the three subscales and the overall GER-MAUQ had strong internal consistency. Cronbach alpha of the overall questionnaire was 0.966, which indicates excellent reliability of the GER-MAUQ.^38^ The Cronbach alpha values of the subscales: ease of use (five items, GER-MAUQ_E), interface and satisfaction (seven items, GER-MAUQ_I) and usefulness (six items, GER-MAUQ_U) showed high internal consistency as well. Their values were 0.814, 0.910 and 0.909, respectively. The reliability analysis was reexamined by using the value of 4 for missing data. Both imputation techniques offer similar values, as presented in Table A2 in the Appendix.

**Table 5.** Cronbach alpha values for the subscales and the overall score of the GER-MAUQ.

Scale^a^	n of items	Cronbach α	Valid n	Excluded^b^	Total
Ease of use	5	0.814	42	11	53
Interface and satisfaction	7	0.910	32	21	53
Usefulness	6	0.909	20	33	53
Overall score	18	0.966	16	37	53

^a^The scales refer to the German translation of the mHealth App Usability Questionnaire.

^b^Listwise deletion based on all variables in the procedure.

#### Construct validity

**Figure 2.**
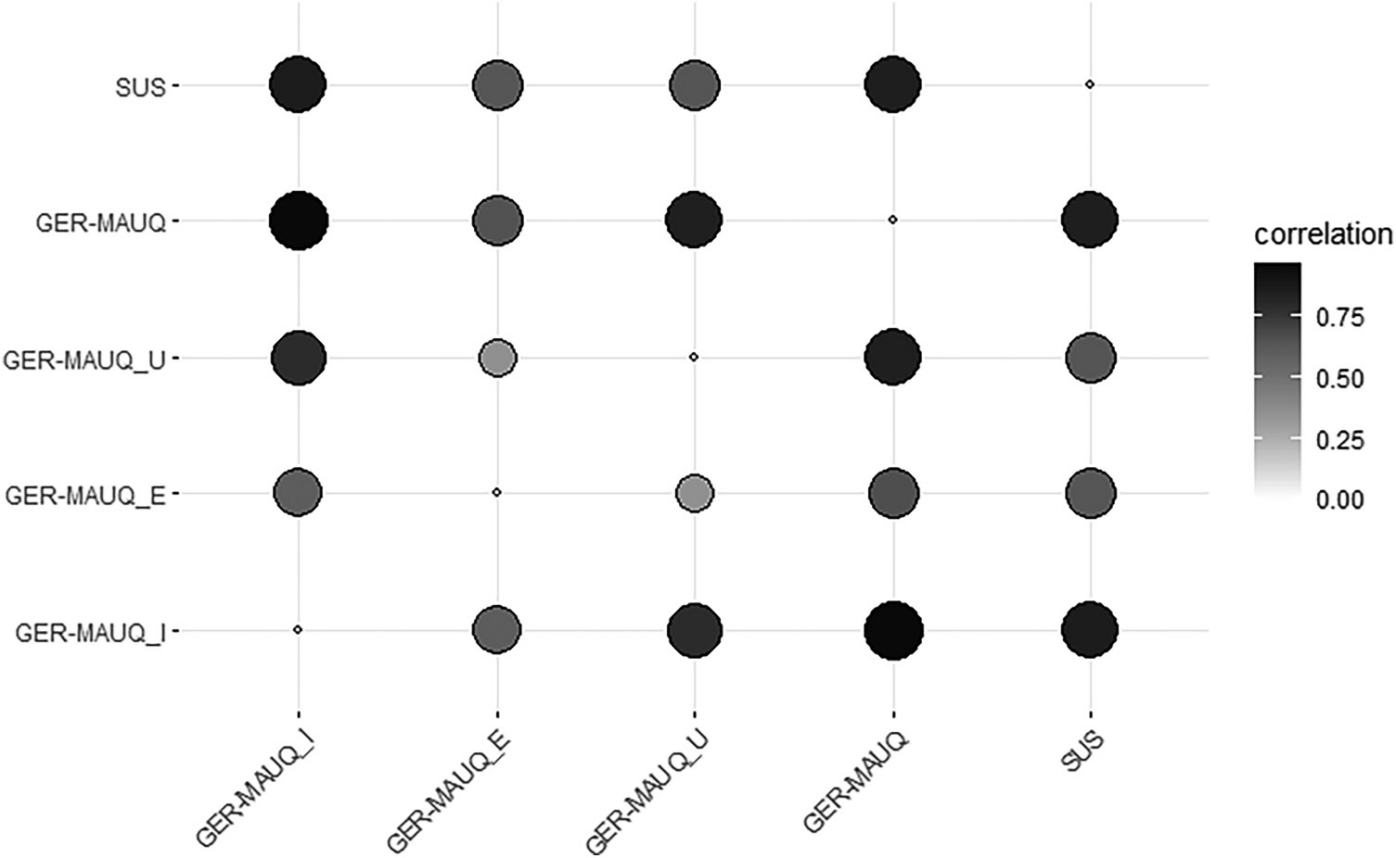
Graphic presentation of the correlation coefficients among the GER-MAUQ overall score, the scores of the three GER-MAUQ subscales and the score of the SUS.

To evaluate the construct validity of the GER-MAUQ, a Pearson or Spearman correlation analysis was conducted, depending on the normal distribution of the data. The scores of the three subscales of the GER-MAUQ, the overall score of the GER-MAUQ and the score of the SUS questionnaire were correlated with each other. Table 6 and Figure 2 show the correlation coefficients and the significance of the respective scores of the GER-MAUQ and the SUS, as well as the respective 95% confidence interval.

**Table 6.** Correlation coefficients among the GER-MAUQ overall score, the scores of the three GER-MAUQ subscales and the score of the SUS (n = 53).

Scale	GER-MAUQ_E^a^	GER-MAUQ_I	GER-MAUQ_U	GER-MAUQ	German SUS
GER-MAUQ_E^a^					
r^b^ (95% CI)	1	0.602 (0.389–0.754)	0.355 (0.086–0.576)	0.665 (0.474–0.796)	0.642 (0.443–0.781)
P value^c^	-	<0.001	0.009	<0.001	<0.001
GER-MAUQ_I					
r^b^		1	0.796 (0.671–0.878)	0.967 (0.943–0.981)	0.866 (0.777–0.921)
P value^c^		-	<0.001	<0.001	<0.001
GER-MAUQ_U					
r^b^			1	0.850 (0.752–0.911)	0.643 (0.452–0.778)
P value^c^			-	<0.001	<0.001
GER-MAUQ					
r^b^				1	0.854 (0.759–0.913)
P value^c^				-	<0.001
German SUS					
r^b^					1
P value^c^					-

GER-MAUQ: German translation of the mHealth App Usability Questionnaire, overall score; GER-MAUQ_E: German translation of the mHealth App Usability Questionnaire, subscale: ease of use; GER-MAUQ_I: German translation of the mHealth App Usability Questionnaire, subscale: interface and satisfaction; GER-MAUQ_U: German translation of the mHealth App Usability Questionnaire, subscale: usefulness; SUS: System Usability Scale.

^a^ Non-normal distribution of MAUQ_E. Spearman correlation coefficients were calculated.

^b^ Correlation coefficient with 95% confidence interval. For normal distribution data Pearson correlation coefficients and for non-normal distribution data Spearman correlation coefficients were calculated. All correlations are significant at the 0.01 level.

^c^ Two-tailed P value.

The results of the analysis indicated that all three subscales correlate significantly with the overall score of the GER-MAUQ (P < 0.05 in all cases). The correlation coefficients among the subscales: ease of use, interface and satisfaction, and usefulness, and the overall score of the GER-MAUQ were 0.665, 0.967 and 0.850, respectively. In addition, the SUS score was strongly correlated with all three subscales of the GER-MAUQ, as the correlation coefficients show: 0.642, 0.866 and 0.643 (P < 0.001 in all cases). The overall score of the GER-MAUQ correlated strongly with the SUS score (r = 0.854). Overall, the correlation analysis of the respective scores indicated criterion and construct validity. We verified the correlation analysis by replacing missing data with the value of 4. Table A3 in the Appendix demonstrates the corresponding correlation coefficients. Both calculations show similar values and underline the concurrent validity of the GER-MAUQ.

### Impact of the demographic characteristics

Table 7 presents the demographic characteristics which have an impact on the GER-MAUQ items. An analysis with the Mann-Whitney U test demonstrated that the utilization period of the app affected item 17 (“I could use the app even when the Internet connection was poor or not available.”, P = 0.002). Participants who used the app between five and seven days answered item 17 higher (median 6) than participants who used the app between two and four days (median 3). Individuals with chronic cardiovascular diseases answered item 18 (“This app provided an acceptable way to access information materials, perform self-assessment and track my own activities.”) significantly differently than people without chronic cardiovascular diseases (P = 0.007). Participants with a chronic disease considered the offer of the mHealth app (item 18) to be less helpful and rated the question with a lower score (median 5) compared to participants without a disease (median 7). 

**Table 7.** Impact of demographic characteristics on GER-MAUQ items: presentation of the significant results (P < 0.05) of the Mann-Whitney U test.

Mann-Whitney U test				Descriptive statistics of both groups			
Characteristics^a^	n	U	P value^b^	Group		n	Median
Chronic disease							
Item 18	52	85.000	0.007	Group 1	With chronic disease	43	5.00
				Group 2	Without chronic disease	9	7.00
No. of days using HerzFit app^c^							
Item 17	25	14.000	0.002	Group 1	2–4 days	7	3.00
				Group 2	5–7 days	18	6.00

^a^Only the characteristics that had a significant impact on an item of the GER-MAUQ in the Mann-Whitney U test are presented.

^b^Two-tailed P value.

^c^Number of days the participants used the HerzFit app in the previous seven days. The HerzFit app is the app that was chosen to validate the German translation of the mHealth App Usability Questionnaire.

The Kruskal-Wallis test showed that the answer to item 4 (“The interface of the app allowed me to use all the functions offered by the app (such as entering information, responding to reminders, viewing information).”) was significantly influenced by the place of residence (P = 0.031). The Bonferroni correction showed that compared to living in a large town (20,000–100,000 inhabitants), living in a village (<5000 inhabitants) had a statistically significant impact (P = 0.042) on item 4. Calculations of means showed that village residents tended to rate item 4 lower (median 3) than residents of a large town (median 5.5). The type of employment was shown to have an impact on the response behavior of item 9 (“I feel comfortable using this app in social settings (in the presence of other people, for example, in the waiting room at the doctor's or in the gym).”, Kruskal-Wallis test P = 0.028). In the pairwise comparison, the difference between employed and retired persons was statistically significant (P = 0.044). Employed persons (median 6) rated question 9 higher than retired persons (median 5).

 None of the demographic information had a statistically significant effect on the overall score of the GER-MAUQ (P > 0.05 in all cases), as shown in Tables A4 and A5 in the Appendix.

The results also showed that the behavior of the participants in preferring apps or analog option had no statistically significant impact on the individual items on the GER-MAUQ (P > 0.05 in all cases). This also applies to the participants’ previous usage of a technical device for measuring physical activity (P > 0.05 in all cases). Tables A6 and A7, which can be found in the Appendix, present the results of the Mann-Whitney U test.

## Discussion

### Principal results

This study aimed to translate the patient version of the mHealth App Usability Questionnaire for standalone apps from English into German and to validate it with a well-known, commonly used questionnaire. The results of the psychometric analysis demonstrated that the translated questionnaire GER-MAUQ is a reliable and valid measurement tool to assess the usability of mHealth apps from the patients’ perspective. The GER-MAUQ represents a novel standardized German questionnaire to assess the usability of mHealth apps from the patients’ perspective. In the future, it could be used by researchers and developers of mHealth apps to identify usability problems easily, quickly, and cost-effectively.

A total of 53 of 57 participants responded to the online survey, which means a very low dropout rate compared to other online surveys in the field of eHealth research and indicates a high usability efficiency of the study design.^42^ The item analysis showed that the mean values of the low and high-score groups were well distinguishable from each other. These statistically significant results applied to all items except item 4. Therefore, discussion on the possible removal of item 4 from the GER-MAUQ was deemed necessary. In the correlation analysis, we saw that item 4 was positively and statistically significantly correlated with the overall score of the GER-MAUQ. After comparison with other studies in which the MAUQ was utilized, and according to the results of the correlation analysis, we decided to retain item 4. We believe that the statement helps to measure ease of use and is therefore important for the evaluation of usability.

A data analysis proved the high reliability and validity of the GER-MAUQ. The values for Cronbach alpha for the entire questionnaire and for the three subscales: ease of use, interface and satisfaction, and usefulness, were high (0.966, 0.814, 0.910, and 0.909), indicating strong internal consistency. Furthermore, a correlation analysis clearly showed that the scores of the subscales, the overall score of the GER-MAUQ and the score of the SUS highly correlated with each other. The correlation coefficient of the SUS and the GER-MAUQ overall score was r = 0.854, and the coefficients of the subscales and the SUS were r = 0.642, r = 0.866, and r = 0.643, proving good criterion and construct validity. Usability studies of mobile applications in the areas of diabetes and cardiology, with populations comparable to those of our study cohort, have shown similar results implicating that positive effects could be expected in larger cohorts.^43,44^ Validation in a larger population is nonetheless recommended. Overall, the analysis demonstrated that the translated questionnaire GER-MAUQ is a reliable and valid measurement tool for evaluating the usability of standalone mHealth apps from the patients’ point of view.

The results of this study are similar to those of the development and validation study of the English MAUQ. In both studies, the values of Cronbach alpha for the scales and subscales of the MAUQ and GER-MAUQ showed high internal consistency: for MAUQ 0.914 and for GER-MAUQ 0.966. Also regarding the examination of construct validity, correlation coefficients were calculated between the subscales and scales of the MAUQ and the SUS. In the analysis of the original study, the correlation coefficient between the total score of the MAUQ and the SUS was 0.7168, and in the data analysis of the GER-MAUQ, it was 0.854. In the development study, validity was also analyzed using the Post-Study System Usability Questionnaire. Since there is no validated German version of the questionnaire in the literature, it was not used in this study. In the original study, an exploratory factor analysis was conducted. The results showed a division into three factors. Due to the small number of cases, we did not perform a factor analysis.^19^

### Overview of the current state of research

As literature research indicates, there was previously no valid questionnaire that specifically measured the usability of mHealth apps from the perspective of patients and providers. Zhou et al. recognized the need for and developed a tool specifically for this purpose.

The ISONORM 9241-110-S^23^ and ISOMetrics^24^ questionnaires are well-known German-language measurement instruments for evaluating usability. The ISONORM 9241-110-S consists of 21 questions, each with a positive and a negative statement, which are to be evaluated on a seven-point scale from very negative to very positive. The time required is 5–10 min. The questionnaire has been examined for reliability and validity and has a high internal consistency.^23,45^ ISONorm is not recommended for a survey with patients or target users of mHealth apps, because the wording of the questions is difficult to understand for them. The ISOmetrics questionnaire evaluates the usability of an interactive software system. The questionnaire is divided into seven subscales and consists of a five-point scale. It is characterized by simple usage and high validity. A major disadvantage of the questionnaire is the large amount of time required. The long version takes at least 2 h and the short version 30 to 60 min.^24^

The Health-ITUES questionnaire is another usability evaluation tool for health information technology.^46^ The questionnaire contains 20 items in the categories: quality of work, perceived usefulness, perceived ease of use, and user control. Nevertheless, questions must be adapted according to the user, which is difficult for researchers without experience in the field of questionnaire development. Likewise, a valid German translation has not yet been undertaken.

The widely-used Mobile Application Rating Scale (MARS) assesses the quality of mHealth apps and contains usability components.^47^ The MARS contains 23 items that assess the quality of mobile health apps in five categories: engagement, functionality, aesthetics, information quality, and subjective quality. A validated German version of the MARS exists; however, this version was designed for health professionals, such as researchers, clinicians, and experts in the field of mHealth, not specifically for patients.^48^ A precondition for an assessment using the MARS is that the user has expertise in the field of mHealth and is trained in the use of MARS items and subscales. With this in the mind, the user version uMARS was developed and validated in English.^49^ As yet, there is no German translation of this user version.

The MARS and the uMARS are the most used questionnaires for measuring the quality of mHealth apps, while the SUS, the PSSUQ and the Computer System Usability Questionnaire (CSUQ) are the most frequently used questionnaires for usability assessment.^16^ The PSSUQ and CSUQ are similar and have been designed to assess user satisfaction with the system usability.^15^ It is striking that the MAUQ is used less frequently compared to other questionnaires, even though it was designed specifically for use with mHealth apps. One explanation for this could be that the MAUQ was published just four years ago. Our translation into German could boost the usefulness of the MAUQ and draw more attention. Following this aim, our work presents a novel version of a German-language questionnaire for mHealth app usability assessment from the patient's perspective.

### Limitations

Our validation study used only one mHealth application. The test period lasted seven days. As a result of both these limiting factors, not all participants could answer several of the GER-MAUQ questions on the Likert scale. For example, only 25 participants answered item 17, which asked whether the app could be used even when the Internet connection was poor or not available. The large amount of missing data led to a lower test power in the statistical analysis. Thus, it cannot be ruled out that the results of the validation would have been different with a different app or a longer test period.

Participants in the study were recruited through channels including the German Heart Foundation, the German Hypertension League and social media. Therefore, we assumed that the participants were interested in dealing with their own health and possibly take more time to use the app than other people. The participant demographic characteristics also differ from those of the overall German population. More than 80% of the participants were over 50 years old and more than 90% had previous experience in measuring their physical activity. In addition, more than half of the participants had the highest school-leaving certificate, indicating a higher level of general education among the participants than the average for the population at large. The evaluation showed that individual characteristics of the participants had a statistically significant impact on response to individual questions of the GER-MAUQ. This concerned items 4, 9, 17 and 18. Researchers who use the GER-MAUQ in the future should take this into account in their response evaluation.

For validation of the GER-MAUQ, only a small sample size of 53 participants was available. With a larger cohort, further analyses, such as conducting a factor analysis, could have been performed. Nevertheless, the number of participants was sufficient for calculating the correlation coefficients between the scores of the overall GER-MAUQ, the subscales, and the SUS. Since the results of the psychometric analysis clearly confirmed the validity of the GER-MAUQ, it was not necessary to conduct a factor analysis.^50^ In the future, a wider base of data should be collected to confirm the three dimensions according to the subscales, via a factor analysis in order to strengthen the dimensionality analysis. Taking known criteria for development and validation of questionnaires into account^50–52^ it should be kept in mind that our sample size is small and results of inter-item and item-total correlations highly depend on the analysis performed in the development study. Furthermore, item reduction analysis was skipped in order to keep the transferability of the results of the initial English MAUQ version to the current study. It could be possible that some items in the German translation were considered as redundant and therefore, were not answered.

Furthermore, we were not able to perform a test-retest analysis since study participants were no longer able to use the same app version and results of the retest would have been biased.

Additionally, preliminary psychometric testing of the pre-final version of the translated instrument with a bilingual sample was not conducted as this would have further reduced the sample size. However, it is not always performed and thus, justifiable to be omitted.^53^

Eventually, we decided to count the “not to answer” response option as missing data which implies our assumption that data are not missing at random.^54^ Since the original validation study of the MAUQ used the value 4 for missing, we supplemented the statistical analysis and performed further tests, checking the results with and without imputation. Nonetheless, it cannot be ruled out that by deciding against multiple imputation we added bias to the analysis.

## Conclusions

This study presents a novel German validated version of the mHealth App Usability Questionnaire for patients, thus enabling a standardized assessment of the usability of standalone mHealth apps for patients in Germany. The data analysis proved that the translated questionnaire GER-MAUQ is a reliable and valid measurement tool for assessing the usability of standalone mHealth apps from the patients’ perspective. As the validation relies on results from patients with cardiovascular disease, further research with a larger sample and other samples is recommended. Regarding the increasing impact of digital transformation in German healthcare and growing requirements for fast successful implementation of mHealth applications, the GER-MAUQ offers an effective tool for assessment and evaluation of mHealth apps’ usability.

## Multimedia Appendix

**Table A1.** Descriptive statistics of the items and scale values for the GER-MAUQ and SUS; missing data were replaced with the value of 4.

	n^a^	Median (range)	Mean (SD)
Items^b^			
1	53	5 (1–7)	5.06 (1.726)
2	53	6 (1–7)	5.28 (1.823)
3	53	6 (1–7)	5.58 (1.715)
4	53	5 (1–7)	4.81 (1.991)
5	53	4 (1–7)	4.47 (2.118)
6	53	5 (1–7)	4.55 (1.976)
7	53	5 (1–7)	4.40 (1.905)
8	53	5 (1–7)	4.47 (1.887)
9	53	5 (1–7)	4.77 (1.794)
10	53	6 (1–7)	5.13 (1.819)
11	53	5 (1–7)	4.60 (2.332)
12	53	5 (1–7)	4.23 (2.006)
13	53	5 (1–7)	4.62 (2.132)
14	53	4 (1–7)	3.23 (1.977)
15	53	4 (1–7)	3.62 (1.934)
16	53	3 (1–7)	3.28 (1.905)
17	53	4 (1–7)	4.53 (1.552)
18	53	5 (1–7)	4.79 (1.843)
Scale			
GER-MAUQ_E	53	5.2 (1–7)	5.042 (1.399)
GER-MAUQ_I	53	4.857 (1–7)	4.593 (1.526)
GER-MAUQ_U	53	4 (1–6.5)	4.013 (1.424)
GER-MAUQ	53	4.556 (1.17- 6.7)	4.524 (1.283)
German SUS	53	70 (22.5–97.5)	67.5 (18.779)

GER-MAUQ: German translation of the mHealth App Usability Questionnaire, overall score; GER-MAUQ_E: German translation of the mHealth App Usability Questionnaire, subscale: ease of use; GER-MAUQ_I: German translation of the mHealth App Usability Questionnaire, subscale: interface and satisfaction; GER-MAUQ_U: German translation of the mHealth App Usability Questionnaire, subscale: usefulness; SD: standard deviation; SUS: System Usability Scale.

^a^ Number of participants who rated item on the Likert scale.

^b^ Items 1–18 represent the 18 items of the German translation of the mHealth App Usability Questionnaire.

**Table A2.** Cronbach alpha values for the subscales and the overall score of the GER-MAUQ. Missing data were replaced with the value of 4.

Scale^a^	n of items	Cronbach α	Valid n	Excluded^b^	Total
Ease of use	5	0.798	53	0	53
Interface and satisfaction	7	0.890	53	0	53
Usefulness	6	0.845	53	0	53
Overall score	18	0.927	53	0	53

^a^ The scales refer to the German translation of the mHealth App Usability Questionnaire.

^b^ Listwise deletion based on all variables in the procedure.

**Table A3.** Correlation coefficients among the GER-MAUQ overall score, the scores of the three GER-MAUQ subscales and the score of the SUS (n = 53). Missing data were replaced with the value of 4.

Scale	GER-MAUQ_E	GER-MAUQ_I	GER-MAUQ_U	GER-MAUQ	German SUS
GER-MAUQ_E^a^					
r^b^	1	0.612 (0.402–0.760)	0.385 (0.119–0.598)	0.678 (0.493–0.804)	0.637 (0.437–0.777)
P value^c^	-	<0.001	0.004	<0.001	<0.001
GER-MAUQ_I					
r^b^		1	0.821 (0.708–0.893)	0.966 (0.942–0.981)	0.862 (0.772–0.918)
P value^c^		-	<0.001	<0.001	<0.001
GER-MAUQ_U					
r^b^			1	0.873 (0.789–0.925)	0.650 (0.461–0.783)
P value^c^			-	<0.001	<0.001
GER-MAUQ					
r^b^				1	0.850 (0.753–0.911)
P value^c^				-	<0.001
German SUS					
r^b^					1
P value^c^					-

GER-MAUQ: German translation of the mHealth App Usability Questionnaire, overall score; GER-MAUQ_E: German translation of the mHealth App Usability Questionnaire, subscale: ease of use; GER-MAUQ_I: German translation of the mHealth App Usability Questionnaire, subscale: interface and satisfaction; GER-MAUQ_U: German translation of the mHealth App Usability Questionnaire, subscale: usefulness; SUS: System Usability Scale.

^a^ Non-normal distribution of MAUQ_E. Spearman correlation coefficients were calculated.

^b^ Correlation coefficient with 95% confidence interval. For normal distribution data Pearson correlation coefficients and for non-normal distribution data Spearman correlation coefficients were calculated. All correlations are significant at the 0.01 level.

^c^ Two-tailed P value.

**Table A4.** Impact of demographic characteristics on the GER-MAUQ overall score: presentation of the results of the Mann-Whitney U test and their descriptive statistics (n = 53).

Mann-Whitney U test (n = 53)			Descriptive statistics of both groups			
Characteristics	U	P value^a^	Group		n	Median
Gender	322.500	0.612	Group 1	Female	26	4.688
			Group 2	Male	27	4.643
Behavior in using apps or analog options	284.500	0.308	Group 1	Preference for using apps	22	4.941
			Group 2	Case-by-case decision	31	4.333
Previous usage of a technical device for measuring physical activity	96.000	0.465	Group 1	Previously used a technical device	48	4.544
			Group 2	Never used a technical device	5	5.294
Chronic cardiovascular disease	153.000	0.286	Group 1	With disease	44	4.544
			Group 2	Without disease	9	5.294

^a^ Two-tailed P value.

**Table A5.** Impact of demographic characteristics on the GER-MAUQ overall score: presentation of the results of the Kruskal-Wallis test.

Characteristics	Kruskal-Wallis H	P value
Age	7.968	0.240
Marital status	1.169	0.883
Highest level of education	3.008	0.390
Highest professional qualification	1.756	0.882
Place of residence	2.179	0.536
Employment	2.107	0.551
Number of days using the HerzFit app^a^ in the previous 7 days	4.342	0.114

^a^ Number of days the participants used the HerzFit app in the previous seven days. The HerzFit app is the app that was chosen to validate the German translation of the mHealth App Usability Questionnaire.

**Table A6.** Impact of the participants’ behavior in using apps or analog options on each item of the German mHealth App Usability Questionnaire. Presentation of the results of the Mann-Whitney U test. No participant prefers using analog options. Participants were divided into two groups: Group 1 prefers using apps and Group 2 decides case-by-case.

	U	P value^a^
Items^b^		
1	271.000	0.197
2	277.500	0.354
3	318.500	0.890
4	313.500	0.977
5	246.500	0.899
6	256.000	0.252
7	266.500	0.262
8	273.500	0.733
9	131.000	0.279
10	244.500	0.105
11	240.500	0.194
12	280.000	0.348
13	303.000	0.486
14	160.500	0.101
15	263.000	0.280
16	295.000	0.919
17	64.500	0.833
18	306.500	0.657

^a^ Two-tailed P value.

^b^ Items 1–18 represent the 18 items of the German translation of the mHealth App Usability Questionnaire.

**Table A7.** Impact of the previous usage of a technical device for measuring physical activity on each item of the German mHealth App Usability Questionnaire. Presentation of the results of the Mann-Whitney U test. Participants were divided into two groups: Group 1 had previously used a technical device and Group 2 had never used one.

Characteristics	U	P value^a^
Items^b^		
1	84.000	0.264
2	86.000	0.311
3	102.500	0.623
4	84.000	0.317
5	74.500	0.705
6	107.000	0.797
7	106.500	0.728
8	78.000	0.283
9	53.500	0.529
10	106.000	0.715
11	94.500	0.548
12	114.000	0.912
13	89.500	0.345
14	47.000	0.523
15	62.500	0.092
16	104.500	0.792
17	40.500	0.908
18	99.500	0.569

^a^ Two-tailed P value.

^b^ Items 1–18 represent the 18 items of the German translation of the mHealth App Usability Questionnaire.
